# Outcomes on safety and efficacy of left atrial appendage occlusion in end stage renal disease patients undergoing dialysis

**DOI:** 10.1007/s40620-020-00774-5

**Published:** 2020-07-10

**Authors:** Simonetta Genovesi, Luca Porcu, Giorgio Slaviero, Gavino Casu, Silvio Bertoli, Antonio Sagone, Monique Buskermolen, Federico Pieruzzi, Giovanni Rovaris, Alberto Montoli, Jacopo Oreglia, Emanuela Piccaluga, Giulio Molon, Mario Gaggiotti, Federica Ettori, Achille Gaspardone, Roberto Palumbo, Francesca Viazzi, Marco Breschi, Maurizio Gallieni, Gina Contaldo, Giuseppe D’Angelo, Pierluigi Merella, Fabio Galli, Paola Rebora, Mariagrazia Valsecchi, Patrizio Mazzone

**Affiliations:** 1 Dipartimento di Medicina e Chirurgia-School of Medicine and Surgery, Università di Milano-Bicocca-University of Milano-Bicocca, Via Cadore 48, 20900 Monza, MB Italy; 2 Nephrology Unit, San Gerardo Hospital, Monza, Italy; 3 Laboratory of Methodology for Clinical Research, Oncology Department, Istituto di Ricerche Farmacologiche IRCCS Mario Negri, Milan, Italy; 4 Nephrology Unit, IRCCS Ospedale San Raffaele, Milan, Italy; 5 San Francesco Hospital, Nuoro. ATS Sardegna Nuoro, Nuoro, Italy; 6 Dialysis and Nephrology Unit-IRCCS-Multimedica, Sesto S.Giovanni, Italy; 7 Electrophysiology Unit-IRCCS-Multimedica, Sesto S.Giovanni, Italy; 8 Department of Nephrology and Dialysis, Luigi Sacco Hospital, Milan, Italy; 9 Interventional Electrophysiology Unit, San Gerardo Hospital, Monza, Italy; 10 Nephrology Unit, Niguarda Hospital, Milan, Italy; 11 Interventional Cardiology Unit, Niguarda Hospital, Milan, Italy; 12 Cardiology Department, IRCCS Sacro Cuore Don Calabria Hospital, Negrar, Italy; 13 Nephrology Unit, ASST degli Spedali Civili di Brescia, Brescia, Italy; 14 Cardiology Unit, ASST degli Spedali Civili di Brescia, Brescia, Italy; 15 Cardiology Unit, S.Eugenio Hospital, Rome, Italy; 16 Nephrology Unit, S.Eugenio Hospital, Rome, Italy; 17 Nephrology Unit, San Martino-IST, Genoa, Italy; 18 Cardiology Unit, USL Toscana Sud-Est, Grosseto, Italy; 19 Cardiac Pacing Unit, IRCCS Ospedale San Raffaele, Milan, Italy

**Keywords:** Atrial fibrillation, Bleeding, Dialysis, Left atrial appendage, Thromboembolism

## Abstract

**Background:**

In patients with end stage renal disease and atrial fibrillation (AF), undergoing chronic dialysis, direct oral agents are contraindicated and warfarin does not fully prevent embolic events while increasing the bleeding risk. The high hemorrhagic risk represents the main problem in this population. Aim of the study was to estimate the safety and efficacy for thromboembolic prevention of left atrial appendage (LAA) occlusion in a cohort of dialysis patients with AF and high hemorrhagic risk.

**Methods:**

Ninety-two dialysis patients with AF who underwent LAA occlusion were recruited. For comparative purposes, two cohorts of dialysis patients with AF, one taking warfarin (oral anticoagulant therapy, OAT cohort, n = 114) and the other not taking any OAT (no-therapy cohort, n = 148) were included in the study. Primary endpoints were (1) incidence of peri-procedural complications, (2) incidence of 2-year thromboembolic and hemorrhagic events, (3) mortality at 2 years. In order to evaluate the effect of the LAA occlusion on the endpoints with respect to the OAT and No-therapy cohorts, a multivariable Cox regression model was applied adjusted for possible confounding factors.

**Results:**

The device was successfully implanted in 100% of cases. Two major peri-procedural complications were reported. No thromboembolic events occurred at 2-year follow-up. The adjusted multivariable Cox regression model showed no difference in bleeding risk in the OAT compared to the LAA occlusion cohort in the first 3 months of follow-up [HR 1.65 (95% CI 0.43–6.33)], when most of patients were taking two antiplatelet drugs. In the following 21 months the bleeding incidence became higher in OAT patients [HR 6.48 (95% CI 1.32–31.72)]. Overall mortality was greater in both the OAT [HR 2.76 (95% CI 1.31–5.86)] and No-Therapy [HR 3.09 (95% CI 1.59–5.98)] cohorts compared to LAA occlusion patients.

**Conclusions:**

The study could open the way to a non-pharmacological option for thromboembolic protection in dialysis patients with AF and high bleeding risk.

**Electronic supplementary material:**

The online version of this article (10.1007/s40620-020-00774-5) contains supplementary material, which is available to authorized users.

## Introduction

Non valvular atrial fibrillation (AF) is the most common cardiac arrhythmia in the general population. The main complications of AF are stroke and increased risk of death. Oral anticoagulant therapy (OAT) is the cornerstone for the management of AF patients at high risk of stroke.

Current guidelines suggest treatment with Vitamin K Antagonists (VKAs) or Direct Oral Anticoagulants (DOACs) for stroke prevention in AF patients with a thromboembolic risk score (CHA2DS2VASc score) of at least 1 in males and at least 2 in females [1].

Patients with end stage renal disease (ESRD) undergoing dialysis have a high prevalence and incidence of AF [2]. Moreover, AF is associated to increased mortality in this population [2]. Indeed, VKAs fail to demonstrate a clear benefit for stroke prevention in these patients, and some studies have raised concerns about the possibility that VKAs may generate more harm than benefit [3–5]. Moreover, dialysis patients have a higher risk of bleeding due to platelet function alterations associated with uremia [6]. Increased mortality due to hemorrhagic events has been shown in US dialysis patients taking DOACs [7] and European cardiology guidelines suggest to avoid routine use of DOACs in patient with severe renal dysfunction [8].

In the last years, Left Atrial Appendage (LAA) occlusion has emerged as an alternative option to OAT for AF patients who are at high thromboembolic risk [9] and not suitable for OAT and recent cardiology guidelines state that LAA occlusion should be considered in this subset of patients [10]. Retrospective studies suggest the efficacy of LAA occlusion in reducing thromboembolic risk in patients with chronic kidney disease (CKD), but very few data are available on outcomes in ESRD patients [11–14]. Recently, we reported the design of the study and some preliminary results about feasibility of LAA occlusion in dialysis patients [15]. In the present study, we report the outcomes of a long-term follow-up of a relatively large dialysis population that underwent LAA occlusion.

## Methods

### Study design

This is an Italian, multi-institutional, prospective, open label, observational study performed according to STROBE guidelines. Three independent cohorts were followed and compared: LAA occlusion, oral anticoagulant therapy (OAT) and No-Therapy cohorts. The study design and the sample size of the LAA occlusion cohort was previously published [15].

The study protocol adhered to the 1975 Helsinki Declaration for Ethical Treatment of Human Subjects, with local ethics committee approval (Comitato Etico della Provincia di Monza e Brianza, study LAAO-DIA, 17032016). All involved subjects provided an informed consent to participate and for data publication.

Eligibility criteria were (1) ESRD requiring renal replacement therapy (haemodialysis or peritoneal dialysis) (2) documented AF (paroxysmal, persistent or permanent) (3) CHA2DS2VASc score ≥ 1 in men and ≥ 2 in women and HASBLED score ≥ 3 or contra-indications for long-term anticoagulant treatment (e.g. previous life-threatening bleeding without a reversible cause) (4) age > 18 years and informed consent to participate in the study. Primary outcomes were (1) incidence of peri-procedural complications (stroke, systemic thromboembolism, bleeding, pericardial effusion, displacement of the device, cardiac tamponade and death) within 30 days of the procedure (2) cumulative incidence of 2-year thromboembolic and bleeding events (first event) (3) mortality and cumulative incidence of cardiovascular events (first event) at 2-years. For comparative purposes, two other cohorts of dialysis patients with documented AF, one taking VKAs (OAT cohort) and the other not taking any anticoagulant therapy (No-Therapy cohort) were included in the study. Both cohorts derive from the database of a prospective study previously performed by our group running from October 2010 to December 2014. All selected patients fitted the same inclusion criteria as the LAA occlusion cohort, but had not undergone the procedure [16, 17].

### Data collection and definitions

Data were collected regarding the cause of ESRD, dialysis duration, comorbidities and echocardiographic parameters.

The following comorbidities were collected: arterial hypertension, diabetes mellitus, dyslipidemia, peripheral arterial disease, ischemic heart disease, heart failure, chronic pulmonary disease (see supplementary material for definitions).

The following echocardiography parameters were collected: presence of left ventricular hypertrophy (LVH), left ventricular dysfunction, atrial dilation (see supplementary material for definitions).

Different types of AF were defined in agreement with the European Society of Cardiology (1) (see supplementary material for definitions).

Systemic thromboembolism was collected only if imaging-proven (computed tomographic scan or nuclear magnetic resonance) and major bleeding was defined as a fall in hemoglobin level of 2 g/dl or more or documented transfusion of at least 2 units of packed red blood cells, or an involvement of a critical anatomical site (intracranial, spinal, ocular, pericardial, articular, intramuscular with compartment syndrome, retroperitoneal) [18].

Different types of AF were defined in agreement with the European Society of Cardiology [1]. In all patients, the thromboembolic (CHA2DS2VASc) and hemorrhagic (HASBLED) scores were determined to quantify patient-specific risk of thromboembolic and bleeding events [19].

### Statistical methods

Baseline covariate distributions were summarized using descriptive statistics (median and range for continuous variables, and frequencies for categorical variables). The multinomial logistic regression model was used to detect imbalances between baseline covariate distributions.

Survival distributions were estimated by the Kaplan–Meier method. For the LAA occlusion cohort, all times were calculated from the date of the procedure and the survival status was updated on 31 December 2018. For all cohorts data were right-censored in case of last date of follow-up or patient’s death. Based on the completeness index (C) of follow-up the comparison and treatment effects estimates were limited to the first 2 years of follow-up, and were assessed using the log-rank test and the Cox regression model, respectively.

#### Multivariable Cox regression models

Results of the Cox models are expressed in terms of estimated hazard ratios (HR), 95% confidence intervals (95% CI) and p values. When evaluating first bleeding event as outcome, the proportional hazard assumption was not satisfied; we accounted for that by splitting time in two periods (before and after 3 months), as many of patients who underwent to LAA occlusion were taking two antithrombotic drugs during the first 3 months after the procedure. A backward selection procedure was applied to the multivariable Cox models. The full models used as predictors those variables that were significantly different at the 0.20 level in the univariate analysis comparing the three cohorts (refer to Table 1). Patient cohorts were forced into the multivariable Cox models.

**Table 1:** Clinical characteristics and comorbidities of the study population

	LAA occlusion N = 92	Cohort		Odds ratio		
		OAT N = 114	No therapy N = 148	OAT	No therapy	p value
Gender, N (%)						
Male	71 (77.2)	75 (65.8)	83 (56.1)	0.57	0.38	0.004
Age, N (%)						
Yrs (median [IQR]) > = 75 yrs	74 [76,80] 42 (45.7)	76 [71,80] 64 (56.1)	76 [69, 82] 85 (57.4)	1.03 1.52	1.03 1.61	0.043 0.177
Dialytic age, N (%)						
> = 3 yrs	34 (38.2)	66 (57.9)	85 (57.4)	2.22	2.18	0.007
Missing data	3 (3.3)	0	0			
BMI kg/m^2^						
N	75	97	126	1.00	0.97	0.269
Median	25.0	24.5	23.3			
Min–max	14.0–42.0	3.0–44.0	1.0–49.1			
Current smoking, N (%)						
Yes	6 (6.7)	12 (11.3)	17 (12.9)	1.77	2.04	0.351
Missing data	3 (3.3)	8 (7.0)	16 (10.8)			
CHA2DS2VASc						
Score (median [IQR])	4 [3, 5]	4 [4, 5]	5 [3, 6]	1.36	1.35	0.001
HASBLED						
Score (median [IQR])	4[4, 5]	4 [3, 5]	4[4, 5]	0.66	0.87	0.050
Atrial fibrillation, N (%)						
Permanent	43 (46.7)	60 (52.6)	33 (22.3)	1		< 0.001
Persistent	15 (16.3)	42 (36.8)	72 (48.6)	2.01	6.25	
Paroxysmal	34 (37.0)	12 (10.5)	43 (29.1)	0.25	1.65	
Comorbidities, N (%)						
Hypertension	82 (89.1)	95 (83.3)	131 (88.5)	0.61	0.94	0.368
Diabetes mellitus	33 (35.9)	36 (31.6)	50 (33.8)	0.83	0.91	0.809
Dyslipidemia	49 (53.3)	45 (39.5)	41 (27.7)	0.57	0.34	< 0.001
Peripheral artery disease	50 (54.3)	83 (72.8)	101 (68.2)	2.25	1.81	0.017
Ischaemic heart disease	43 (46.7)	56 (49.1)	75 (50.7)	1.10	1.17	0.839
Heart failure	32 (34.8)	49 (43.0)	54 (36.5)	1.41	1.08	0.419
Ischaemic stroke	9 (9.8)	12 (11.4)	9 (6.7)	1.19	0.66	0.431
Missing data	0 (0)	9 (7.9)	13 (8.8)			
Chronic pulmonary disease	18 (19.6)	21 (18.4)	30 (20.3)	0.93	1.05	0.932
Thromboembolic pulmonary disease	2 (2.2)	1 (0.9)	5 (3.4)	0.40	1.57	0.444
Bleeding	56 (60.9)	15 (13.2)	36 (24.3)	0.10	0.21	< 0.001
Echocardiography, N (%)						
Atrium dilatation	78 (87.6)	na	na	nd	nd	nd
Missing data	3 (3.3)	114 (100)	148 (100)			
Left ventricular ejection fraction < 50%	19 (20.9)	28 (25.9)	38 (28.1)	1.33	1.48	0.467
Missing data	1 (1.1)	6 (5.3)	13 (8.8)			
Left ventricular hypertrophy	44 (57.1)	67 (61.5)	82 (61.2)	1.20	1.18	0.808
Missing data	15 (16.3)	5 (4.4)	14 (9.5)			
Antiplatelet	59 (64.1)	32 (28.1)	104 (70.3)	0.22	1.32	< 0.001
Heparin	31 (33.7)	33 (28.9)	30 (20.7)	0.80	0.51	0.075

*LAA* left atrial appendage, *OAT* oral anticoagulant therapy, *na* not available, *nd* not determined

#### Sensitivity analysis

A propensity score analysis was used to control overfitting. In order to evaluate the effect of the LAA occlusion on different outcomes, with respect to the reference cohorts we created a pseudo-population (that mimics a randomized trial) by the use of (stabilized) inverse probability of treatment (and censoring) weights (IPTW) computed by a multivariable logistic model on the propensity to undergo LAA occlusion. The weighted Cox regression model with robust standard error was applied to the IPTW cohort to assess the effect of LAA occlusion on the different endpoints (see supplementary material, Expanded statistical methods).

Since heparin anticoagulation is not used in PD patients, unlike in HD patients in which the drug is administered during the dialysis session and this could affect the outcomes of the study, we performed a sensitivity analysis (Multivariable Cox regression model) excluding the PD patients (n = 2) from the study population (see supplementary material).

Statistical analysis was generated using SAS software for Windows, version 9.4 (Cary, NC: SAS Institute Inc; 2014). Kaplan–Meier plots were obtained using STATA software for Windows, version 15.1 (StataCorp. 2017. Stata Statistical Software: Release 15. College Station, TX: StataCorp LLC) (see supplementary material for expanded statistical methods).

## Results

Ninety-two consecutive patients who had undergone LAA occlusion in 11 Italian participating centers between May 2014 and December 2018 were enrolled in the study. The reference cohorts were composed of 114 (OAT cohort) and 148 (No-Therapy cohort) patients, respectively. Table 1 shows the clinical characteristics of the three cohorts. LAA occlusion patients were more frequently males than No-Therapy patients and had a shorter dialytic age compared to other cohorts. The prevalence of paroxysmal AF was higher in LAA occlusion compared to OAT cohort, while the prevalence of permanent AF was higher in LAA occlusion than in No-Therapy patients. Moreover, patients who underwent the procedure more often had a previous bleeding and showed higher HASBLED and lower CHA2DS2VASc scores. Antiplatelet prescription was more frequent in LAA occlusion than in OAT patients. Results after IPTW are shown in Supplementary Tables 1 and 2. The median follow-up duration was 1.73 (IQR 0.71–2.58) years for the LAA occlusion cohort and 4.0 (IQR 4.00–4.00) years for both the OAT and No-Therapy cohorts. The C index at 2 years was 67, 98 and 98% for LAA occlusion, OAT and No-Therapy cohorts, respectively.

### Peri-procedural complications

Three types of devices were used: 42 Amplatzer-Amulet (St. Jude Medical Inc., St. Paul, MN, USA), 47 Watchman (Boston Scientific, Plymouth, MN, USA) and 3 LAmbre [Lifetech Scientific (Shenzhen) Co. Ltd., Shenzhen, China]. All devices were implanted successfully. Two non-significant (2.5 and 3 mm) post-procedure para-prosthetic leaks were reported. Two major peri-procedural complications were recorded: a hemorrhagic pericardial effusion leading to cardiac tamponade (LAmbre device) and an acute lower limb ischemia due to rupture of the femoral artery (Watchman device). Furthermore, two hematomas occurred at the vascular access site. No episodes of thrombosis of the device were reported.

### Thromboembolic and hemorrhagic events

**Fig. 1:**
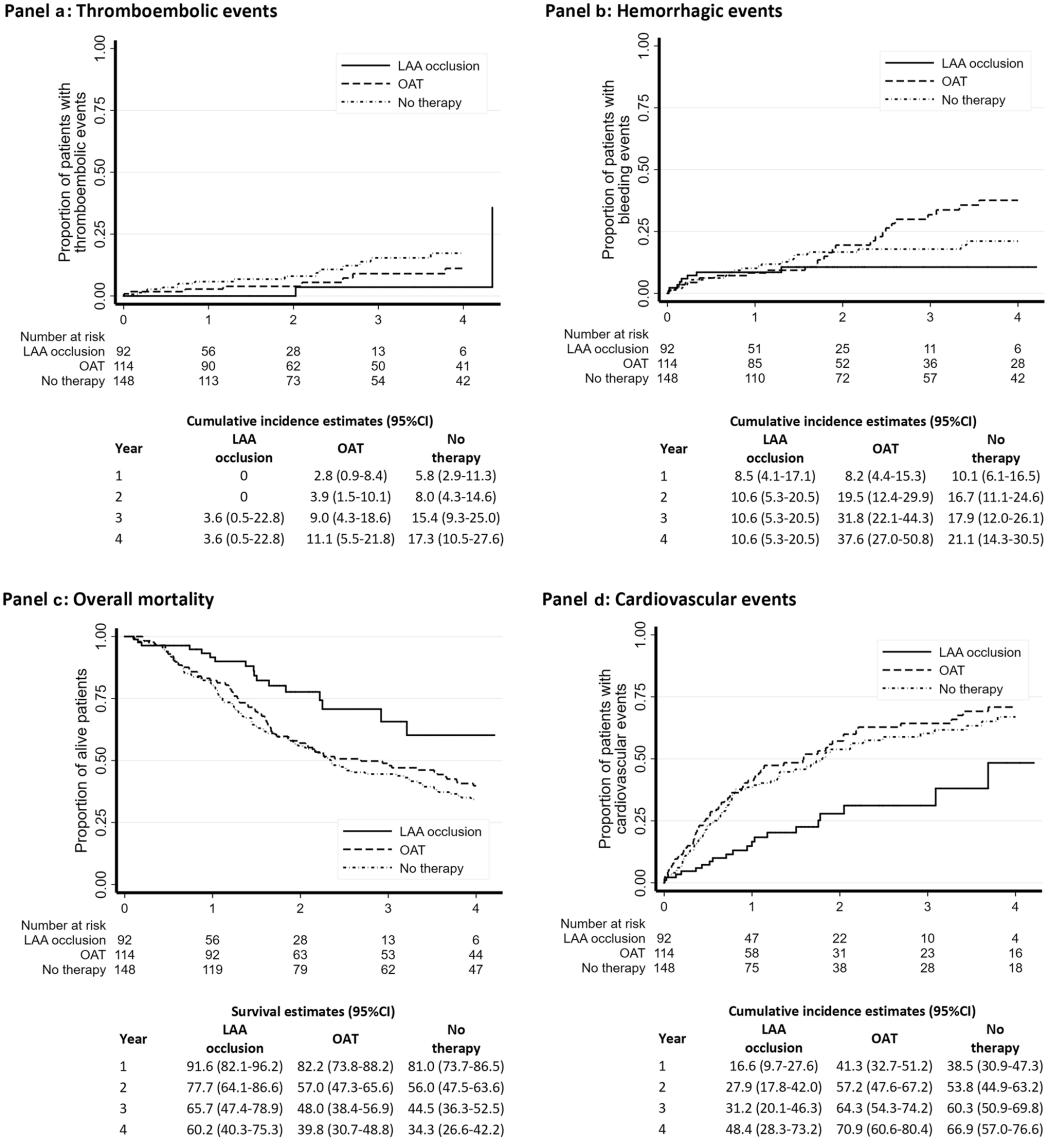
Incidence of thromboembolic (panel **a**) and hemorrhagic (panel **b**) events; and overall survival (panel **c**) and incidence of cardiovascular events (panel **d**), estimated by Kaplan–Meier curves, in the three cohorts of patients

During follow-up, 2 (2.2%), 8 (7.0%), 16 (10.8%) thromboembolic events occurred in the LAA occlusion, OAT and No-Therapy cohorts, respectively. Univariate analysis (Kaplan–Meier curves and log-rank test) showed no difference in the incidence of thromboembolic events in the LAA occlusion vs OAT cohort [2-year estimate of 0 vs 3.9% (95% CI 1.5–10.1); p = 0.092], while the incidence was significantly lower in the LAA occlusion cohort vs the No-Therapy cohort [2-year estimate of 0 vs 8.0% (95% CI 4.3–14.6) p = 0.021] (Fig. 1, panel a).

**Fig. 2:**
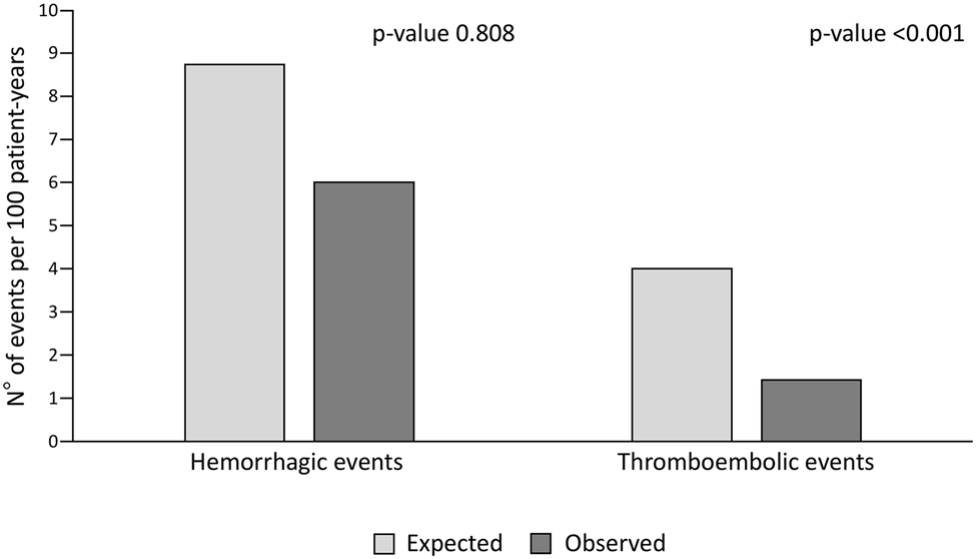
Expected and observed hemorrhagic and thromboembolic events according to CHA2DS2VASc and HASBLED scores in the LAA occlusion cohort

For what regards hemorrhagic events (first event), 8 (8.7%), 27 (23.7%), 24 (16.2%) episodes occurred in the LAA occlusion, OAT and No-Therapy cohorts, respectively. No differences were observed between bleedings in the three cohorts [2-year estimate of 10.6% (95% CI 5.3–20.5) for LAA occlusion, 19.5% (95% CI 12.4–29.9) for OAT and 16.7% (95% CI 11.1–24.6) for No-Therapy; p = 0.474 and p = 0.460 vs LAA occlusion] (Fig. 1, panel b). In the LAA occlusion cohort, 6 out of 8 bleeding episodes occurred in the first 3 months after the procedure. The multivariable Cox regression model for hemorrhagic events did not show any differences among the three cohorts in the first 3 months [HR 1.65 (95% CI 0.43–6.33) OAT vs LAA occlusion and HR 0.57 (95% CI 0.18–1.84) No-Therapy vs LAA occlusion]. However, the risk of bleeding in the following 21 months was significantly higher in both OAT [HR 6.48 (95% CI 1.32–31.72)], and No-Therapy patients [HR 4.87 (95% CI 1.08–21.97)] compared to LAA occlusion patients (Table 2). The sensitivity analysis confirmed the result of the multivariable Cox model, with the exception of the risk of bleeding in months 4–24, which was not different between the LAA occlusion cohort and the No-Therapy cohort (supplementary Table 3). The results were unchanged after excluding PD patients from the study population (supplementary Table 4). The multivariable Cox model was not applied for the evaluation of thromboembolic events as no events occurred in the first 2 years in the LAA occlusion cohort. In the LAA occlusion cohort, the median thromboembolic (CHA2DS2VASc) and hemorrhagic (HASBLED) scores were 4.0 (range 2–8) and 4.0 (range 3–6), respectively and the number of observed events was lower than the number of expected events according to the scores [1.4 (95% CI 0–2.8) vs 4.0/100 patient years for thromboembolism, p < 0.001 and 5.5 (95% CI 0–28.0) vs 8.7/100 patient years for bleeding, p = 0.808] (Fig. 2).

**Table 2:** Cox model on hemorrhagic events, overall mortality and cardiovascular events at 2 years of follow-up

	Univariate analysis			Multivariate analysis*		
	HR	95% CI	p value	HR	95% CI	p value
OAT vs LAA occlusion						
Hemorrhagic events (1–3 months)	0.61	0.19–1.99	0.409	1.65	0.43–6.33	0.464
Hemorrhagic events (4–24 months)	3.45	0.77–15.46	0.105	6.48	1.32–31.72	0.021
				Time-interaction p value:		0.198
Overall mortality	2.22	1.20–4.11	0.011	2.76	1.31–5.86	0.008
Cardiovascular events	2.81	1.64–4.81	< 0.001	5.07	2.49–10.34	< 0.001
No-therapy vs LAA occlusion						
Hemorrhagic events (1–3 months)	0.55	0.18–1.72	0.308	0.57	0.18–1.84	0.350
Hemorrhagic events (4–24 months)	3.68	0.84–16.11	0.083	4.87	1.08–21.97	0.039
				Time-interaction p value:		0.027
Overall mortality	2.40	1.32–4.35	0.004	3.09	1.59–5.98	0.001
Cardiovascular events	2.50	1.47–4.25	0.001	3.11	1.78–5.42	< 0.001

Hemorrhagic events are evaluated in the first 3 months and in the following 21 months from procedure

*LAA* left atrial appendage, *OAT* oral anticoagulant therapy

*Adjusted for gender, age, dialytic age, CHA2DS2VASc, HASBLED, type of atrial fibrillation, dyslipidemia, peripheral artery disease, previous bleeding, antiplatelet, heparin

### Mortality and cardiovascular events

During follow-up, 17 (18.5%), 67 (58.8%), 94 (63.5%) deaths occurred in the LAA occlusion, OAT and No-Therapy cohorts, respectively. Overall survival was significantly higher in LAA occlusion patients compared to the other cohorts [2-year estimate of 77.7% (95% CI 64.1–86.6) for LAA occlusion, 57.0% (95% CI 47.3–65.6) for OAT and 56.0% (95% CI 47.5–63.6) for No-Therapy; LAA occlusion vs OAT p = 0.009 and LAA occlusion vs No-Therapy p = 0.003] (Fig. 1, panel c). Nonfatal cardiovascular events occurred 20 (21.7%), 68 (59.6%), 77(52.0%) times in the LAA occlusion, OAT and No-Therapy cohorts, respectively and their incidence was lower in LAA occlusion patients compared to the other cohorts [2-year estimate of 27.9% (95% CI 17.8–42.0) for LAA occlusion, 57.2% (95% CI 47.6–67.2) for OAT and 53.8% (95% CI 44.9–63.2) for No-Therapy; LAA occlusion vs OAT p < 0.001 and LAA occlusion vs No-Therapy p < 0.001] (Fig. 1, panel d).

In the multivariable Cox model overall mortality was significantly higher in both OAT [HR 2.76 (95% CI 1.31–5.86)] and No-Therapy [3.09 (95% CI 1.59–5.98)] cohorts in comparison with LAA occlusion patients. The risk of nonfatal cardiovascular events was higher in patients taking warfarin or taking no OAT than in patients who had undergone the procedure [HR 5.07 (95% CI 2.49–10.34) OAT vs LAA occlusion and HR 3.11 (95% CI 1.78–5.42) No-Therapy vs LAA occlusion] (Table 2). The sensitivity analysis confirmed all the results of the multivariable Cox models (supplementary Table 3). The results were unchanged after excluding PD patients from the study population (supplementary Table 4).

### Post-procedural antithrombotic therapy

Each center participating in the study was free to choose the post-procedural therapy considered most suitable for the patient. The majority of patients were discharged from the hospital with a two drugs prescription (n = 65, 70.6%), to be taken for 1 (n = 10, 15.4%), 3 (n = 29, 33.8%) or 6 (n = 8, 12.3%) months. The remaining 18 (16.6%) patients continued the therapy with two drugs for more than 6 months. A fair percentage of patients were discharged taking only one drug (n = 21, 22.8%) and, in subjects with a particularly high risk of bleeding, no therapy was prescribed (n = 2, 2.2%). Only one patient took three drugs for a month. Supplementary Table 5 shows the post-procedural therapies taken by patients undergoing LAA occlusion.

## Discussion

The study suggests that LAA occlusion is not only feasible and safe in patients undergoing dialysis, but that, in the long term, it is also associated with a reduction of thromboembolic events compared to non-treated patients, and of haemorrhagic events compared to patients taking OAT. Furthermore, in our population, 2-year survival is significantly higher in the cohort of patients who underwent the procedure and the incidence of nonfatal cardiovascular events is significantly lower, compared to both the OAT and No-Therapy cohorts.

Few data are available about LAA occlusion outcomes in CKD patients. Case reports in ESRD dialysis patients, retrospectively collected, have been described (10–12). A retrospective analysis of patients undergoing the procedure showed that CKD patients, when compared to non-CKD patients, had a greater number of events at follow-up and a higher risk of acute renal failure associated with the procedure [20]. Some additional information was provided by data from a large registry, which included 19 out of 1014 patients with stage 5 CKD, 14 of whom undergoing dialysis [11]. In this analysis, dialysis patients were merged with those with stage 4 and 5 CKD not on dialysis and a significant reduction in stroke and bleeding compared to the expected annual risk was observed. Recently, Gotzmann et al. retrospectively analysed 128 patients and showed that the incidence of mortality, bleeding or thromboembolism was not significantly higher in the subgroup of 33 patients with ESRD [21].

To our knowledge, this is the first prospective, multicenter study designed to evaluate the efficacy and safety of LAA occlusion in a relatively large sample of patients with ESRD. In our study the number of thromboembolic events in LAA occlusion patients is lower compared to the No-Therapy cohort. In the first 2 years of follow-up, no event is observed in patients who underwent the procedure compared to an incidence of 4% in OAT patients and 8% in patients not taking therapy. Furthermore, long-term hemorrhagic events are significantly less frequent in LAA occlusion patients than in both the cohort taking OAT, and the No-Therapy cohort (10.6, 19.5 and 16.7% at 2 years, respectively). Moreover, in LAA occlusion patients, the incidence of thromboembolic events and of bleeding is less than expected based on CHA2DS2VASc and HASBLED scores. The difference in bleeding risk between the LAA occlusion and the OAT cohorts becomes evident by excluding the events occurring in the first 3 months after the procedure from the analysis, when more than 70% of the patients received the prescription of at least two anticoagulant drugs. The therapeutic attitude shown in our study is similar to that reported in non-CKD patients [22], however the excess of early bleeding should lead us to reconsider whether it is really necessary, in patients with such a high risk of bleeding, to take two drugs rather than one after the procedure.In fact, there are many doubts about the use of dual antiplatelet agents in dialysis patient even in the presence of other clinical situations, such as ischemic heart disease [23]. Recently, even among cardiologists, the need to always prescribe double antiplatelet therapy after LAA occlusion has been questioned, and several registry data suggest that single antiplatelet therapy is just as effective as double antiplatelet therapy [24–27].

An unexpected finding is the reduction in mortality and nonfatal cardiovascular events in the LAA occlusion cohort compared to the other two cohorts. A similar finding has been previously observed in the Protect-AF trial [9]. There are several data showing a better survival in dialysis patients with AF taking OAT, compared to those not taking therapy [16, 28]. In our study, LAA occlusion seems to offer an additional survival advantage. In patients who underwent LAA occlusion no deaths due to neither ischemic nor haemorrhagic stroke occurred, but this is not enough to justify such an important advantage in terms of survival in the LAA occlusion cohort. One hypothesis could be that patients undergoing the procedure are followed with particular attention by an interdisciplinary team of physicians (cardiologists and nephrologists), with the consequence that any clinical problem is addressed by both specialists as soon as it becomes evident. If this is true, the study would further underline the importance of close collaboration between cardiologists and nephrologists in treating nephrological patients presenting cardiological diseases.

The number of peri-procedural complications of our patients is relatively low, although not negligible, and comparable to that reported in cardiology populations without renal disease that underwent the procedure [29, 30]. Probably this favourable safety profile is due to the participation in our study only of skilled operators, very confident with the procedure. Though this could constitute a barrier to the spread of LAA occlusion for thromboembolic prevention in dialysis patients, we believe it is very important to consider this option in this population only if a team of experienced cardiologists, who have already performed a large number of procedures, is available. Another problem for the diffusion of the procedure could be its cost. However, a recent study has shown that LAA occlusion proved to be not only cost-effective, but cost saving relative to warfarin and DOACs [31].

Using DOACs in patients with ESRD remains an open problem. An observational study recently suggested an advantage in terms of efficacy and safety of using DOACs in hemodialysis patients with AF [32]. Achieving positive results from CRTs that compare VKAs and DOACs in ESRD patients would be very important, because it would offer a new therapeutic opportunity to the population of dialysis patients with AF. There are currently two ongoing CRTs to test the safety of apixaban in this population (AXADIA trial, NCT02933697 and RENAL-AF trial, NCT02942407). The preliminary results of the RENAL-AF trial have been disappointing. The study was terminated prematurely and showed similar rates of bleeding with apixaban and warfarin [33]. We are waiting for the results of the AXADIA trial, but at present no studies are available to support the hypothesis that DOACs represent an advantage for thromboembolic prevention over warfarin in ESRD patients with AF [34]. LAA occlusion therefore is an option to be taken into consideration for subjects with advanced CKD and particularly high bleeding risk.

Our study has some strengths and some limitations. Strengths are the sample size, the prospective design of the study and the fact that a comparison was made between the population of patients who underwent the procedure and two other populations with similar clinical characteristics. A limitation is that it is not a controlled and randomized study. However, we think that it would not be ethical neither to randomly assign patients with such a high risk of bleeding to OAT treatment nor to assign patients with a high risk of thromboembolic events to a treatment arm without any antithrombotic therapy. Despite the rigorous statistical approach used in the present study, however, we cannot exclude that physicians proposed the LAA occlusion procedure mainly to patients who seemed less frail. If this were the case, however, the result regarding the feasibility, efficacy and safety of the procedure would, in our opinion, remain valid. A Spanish study is currently underway in ESRD patients (WATCH-HD, NCT03446794) which includes two arms, watchman device vs no-therapy. The study is still under recruitment.

In conclusion, our study could open the way to a non-pharmacological option for thromboembolic protection in a fragile category of AF patients, which otherwise would be destined not to take OAT or to take it exposing themselves to a high risk of bleeding.

## Electronic supplementary material

Below is the link to the electronic supplementary material.

Supplementary material 1 (DOCX 33 kb)

Supplementary material 2 (DOCX 34 kb)
