# Correction to: Outcomes on safety and efficacy of left atrial appendage occlusion in end stage renal disease patients undergoing dialysis

**DOI:** 10.1007/s40620-020-00800-6

**Published:** 2020-07-13

**Authors:** Simonetta Genovesi, Luca Porcu, Giorgio Slaviero, Gavino Casu, Silvio Bertoli, Antonio Sagone, Monique Buskermolen, Federico Pieruzzi, Giovanni Rovaris, Alberto Montoli, Jacopo Oreglia, Emanuela Piccaluga, Giulio Molon, Mario Gaggiotti, Federica Ettori, Achille Gaspardone, Roberto Palumbo, Francesca Viazzi, Marco Breschi, Maurizio Gallieni, Gina Contaldo, Giuseppe D’Angelo, Pierluigi Merella, Fabio Galli, Paola Rebora, Mariagrazia Valsecchi, Patrizio Mazzone

**Affiliations:** 1 Dipartimento di Medicina e Chirurgia-School of Medicine and Surgery, Università di Milano-Bicocca-University of Milano-Bicocca, Via Cadore 48, 20900 Monza, MB Italy; 2 Nephrology Unit, San Gerardo Hospital, Monza, Italy; 3 Laboratory of Methodology for Clinical Research, Oncology Department, Istituto di Ricerche Farmacologiche IRCCS Mario Negri, Milan, Italy; 4 Nephrology Unit, IRCCS Ospedale San Raffaele, Milan, Italy; 5 San Francesco Hospital, Nuoro. ATS Sardegna Nuoro, Nuoro, Italy; 6 Dialysis and Nephrology Unit-IRCCS-Multimedica, Sesto S.Giovanni, Italy; 7 Electrophysiology Unit-IRCCS-Multimedica, Sesto S.Giovanni, Italy; 8 Department of Nephrology and Dialysis, Luigi Sacco Hospital, Milan, Italy; 9 Interventional Electrophysiology Unit, San Gerardo Hospital, Monza, Italy; 10 Nephrology Unit, Niguarda Hospital, Milan, Italy; 11 Interventional Cardiology Unit, Niguarda Hospital, Milan, Italy; 12 Cardiology Department, IRCCS Sacro Cuore Don Calabria Hospital, Negrar, Italy; 13 Nephrology Unit, ASST degli Spedali Civili di Brescia, Brescia, Italy; 14 Cardiology Unit, ASST degli Spedali Civili di Brescia, Brescia, Italy; 15 Cardiology Unit, S.Eugenio Hospital, Rome, Italy; 16 Nephrology Unit, S.Eugenio Hospital, Rome, Italy; 17 Nephrology Unit, San Martino-IST, Genoa, Italy; 18 Cardiology Unit, USL Toscana Sud-Est, Grosseto, Italy; 19 Cardiac Pacing Unit, IRCCS Ospedale San Raffaele, Milan, Italy

## Correction to: Journal of Nephrology 10.1007/s40620-020-00774-5

The article Outcomes on safety and efficacy of left atrial appendage occlusion in end stage renal disease patients undergoing dialysis, written by Simonetta Genovesi, Luca Porcu, Giorgio Slaviero, Gavino Casu, Silvio Bertoli, Antonio Sagone, Monique Buskermolen, Federico Pieruzzi, Giovanni Rovaris, Alberto Montoli, Jacopo Oreglia, Emanuela Piccaluga, Giulio Molon, Mario Gaggiotti, Federica Ettori, Achille Gaspardone, Roberto Palumbo, Francesca Viazzi, Marco Breschi, Maurizio Gallieni, Gina Contaldo, Giuseppe D’Angelo, Pierluigi Merella, Fabio Galli, Paola Rebora, Mariagrazia Valsecchi, and Patrizio Mazzone, was originally published electronically on the publisher’s internet portal on 6 June 2020 without open access. With the author(s)’ decision to opt for Open Choice the copyright of the article changed on 10 July 2020 to © The Author(s) 2020 and this article is licensed under a Creative Commons Attribution 4.0 International License (http://creativecommons.org/licenses/by/4.0/), which permits use, sharing, adaptation, distribution and reproduction in any medium or format, as long as you give appropriate credit to the original author(s) and the source, provide a link to the Creative Commons licence, and indicate if changes were made.

The original article has been updated.

