# Supplementary material for: Outcomes on safety and efficacy of left atrial appendage occlusion in end stage renal disease patients undergoing dialysis
Source: J Nephrol. 2020 Jul 10;34(1):63–73. doi: 10.1007/s40620-020-00774-5 (PMC7881969; doi:10.1007/s40620-020-00774-5)
Supplement: Supplementary file 1 — Supplementary material 1 (DOCX 33 kb) [file 40620_2020_774_MOESM1_ESM.docx]

**SUPPLEMENTARY MATERIAL**

**Definitions of variables**

The following comorbidities were collected: arterial hypertension (systolic blood pressure ≥140mmHg and/or diastolic blood pressure ≥90mmHg, or on anti-hypertensive treatment); diabetes mellitus; dyslipidemia (LDL cholesterol ≥130mg/dl or using lipid-lowering medications); peripheral arterial disease; ischemic heart disease (previous hospitalization due to acute coronary syndrome and/or surgical or percutaneous coronary revascularization procedures); heart failure [presence of left ventricular dysfunction (left ventricular ejection fraction, LVEF <50% at ultrasound examination) and/or previous hospitalization due to acute or chronic heart failure], chronic pulmonary disease.

The following echocardiography parameters were collected: presence of left ventricular hypertrophy (LVH) (left indexed ventricular mass [LVMI] >115 g/m2 in men and >95 g/m2 in women), left ventricular dysfunction (left ventricular ejection fraction [LVEF] <50%), atrial dilation (anterior-posterior left atrial diameter >40 mm and/or left atrial volume >34 ml/m2).

Different types of AF were defined in agreement with the European Society of Cardiology. Paroxysmal AF was defined as a self-terminating episode, usually within 48 h but potentially persisting up to 7 days. Persistent AF was defined as an AF episode that either lasted >7 days or required termination by cardioversion, either pharmacological or electrical. Permanent AF was defined as AF lasting >7 days, combined with a joint decision by the patient and clinician to cease further attempts to restore and/or maintain sinus rhythm.

**Expanded statistical methods**

Baseline covariate distributions were summarized using descriptive statistics (median and range for continuous variables, and frequencies for categorical variables). The multinomial logistic regression model was used to detect imbalances between baseline covariate distributions.

Survival distributions were estimated by the Kaplan-Meier method. Median follow-up and its interquartile range (IQR) were estimated with the reverse Kaplan-Meier method (1). For the LAA occlusion cohort, all times were calculated from the date of the procedure and the survival status was updated on 31 December 2018. For all cohorts data were right-censored in case of last date of follow-up or patient’s death. Based on the completeness index (C) [(2) of follow-up the comparison and treatment effects estimates were limited to the first two years of follow-up, and were assessed using the log-rank test and the Cox regression model, respectively.

Multivariable Cox regression models.

Results of the Cox models are expressed in terms of estimated hazard ratios (HR), 95% confidence intervals (95% CI) and p-values. When evaluating first bleeding event as outcome, the proportional hazard assumption was not satisfied; we accounted for that by splitting time in two periods (before and after 3 months), as many of patients who underwent to LAA occlusion were taking two antithrombotic drugs during the first 3 months after the procedure. A backward selection procedure was applied to the multivariable Cox models. The full models used as predictors the patients cohorts and those variables that were significant at the 0.20 level in the univariate analysis (refer to Table 1). Predictors introduced in the full Cox models had to be significant at the 0.20 level to remain in the models. Patient cohorts were forced into the multivariable Cox models.

Sensitivity analysis

A propensity score analysis was used to control overfitting. In order to evaluate the effect of the LAA Occlusion on mortality, hemorrhagic, cardiovascular and thromboembolic risk with respect to the cohort treated with OAT we created a pseudo-population (that mimics a randomized trial) which mitigates the selection bias in treatment assignment at recruitment (3). This pseudo-population was created by the use of (stabilized) inverse probability of treatment (and censoring) weights (IPTW). IPTW patients were computed by a multivariable logistic model on the propensity to undergo LAA Occlusion that included age, gender, dialytic age, type of AF, CHA2DS2-VASc and HAS-BLED scores, dyslipidemia, peripheral artery disease, heart failure, previous bleedings, antiplatelet therapy, LVEF <50%, LVH and the interaction of the last two variables.

In order to evaluate the balance induced by these weights, the confounders among patients under OAT and not in this pseudo-population were compared by standardized differences (4) and Chi-square test. Furthermore, an inverse probability of censoring weight was also applied to account for loss to follow-up and informative censoring due to death when analyzing hemorrhagic, cardiovascular and thromboembolic outcomes. Final weights were computed as the product of the stabilized weights for treatment and censoring. For two patients, weight was also trimmed at a value of 10.

The weighted Cox regression model with robust standard error was applied to the IPTW cohort to assess the effect of LAA Occlusion on the different endpoints (first bleeding event, overall mortality, first cardiovascular event and first thromboembolic event up to two years from recruitment). An analogous procedure was applied to evaluate the effect of the LAA Occlusion with respect to the not treated cohort, in which we considered the same variables for the computation of weights. As the reference cohorts (OAT and No-therapy cohort) were different, stabilized weights also resulted different. In this second comparison, only one patient’s weight was trimmed at 10.

Results of the Cox models are expressed in terms of estimated hazard ratios (HR), 95% confidence intervals (95% CI) and p-values.

When evaluating first bleeding event as outcome the proportional hazard assumption was not satisfied, thus we accounted for that by splitting time in two periods (before and after 3 months), as many of patients who underwent to LAA Occlusion were taking two antithrombotic drugs during the first 3 months after the procedure. In this case we reported the HR with 95%CI for the two periods (1-3 months and more than 3 months from the procedure) and p-value of the interaction between treatment and time period.

Statistical analysis was generated using SAS software for Windows, version 9.4 (Cary, NC: SAS Institute Inc; 2014). Kaplan-Meier plots were obtained using STATA software for Windows, version 15.1 (StataCorp. 2017. Stata Statistical Software: Release 15. College Station, TX: StataCorp LLC).

**References:**

1. Schemper M, Smith TL. A note on quantifying follow-up in studies of failure time. Control Clin Trials. 1996;17(4):343-6.

2. Clark TG, Altman DG, De Stavola BL. Quantification of the completeness of follow-up. Lancet (London, England). 2002;359(9314):1309-10.

3. Hernan MA RJ. Causal Inference: What If. : Boca Raton:Chapman & Hill/CRC; 2020.

4. Austin PC. Balance diagnostics for comparing the distribution of baseline covariates between treatment groups in propensity-score matched samples. Stat Med. 2009;28(25):3083-107.
