# Supplementary material for: Outcomes on safety and efficacy of left atrial appendage occlusion in end stage renal disease patients undergoing dialysis
Source: J Nephrol. 2020 Jul 10;34(1):63–73. doi: 10.1007/s40620-020-00774-5 (PMC7881969; doi:10.1007/s40620-020-00774-5)
Supplement: Supplementary file 2 — Supplementary material 2 (DOCX 34 kb) [file 40620_2020_774_MOESM2_ESM.docx]

**Supplementary Table 1: LAA occlusion versus OAT cohort**

|  | **Unweighted cohorts** | | | | **Weighted cohorts** | | | |
| --- | --- | --- | --- | --- | --- | --- | --- | --- |
|  | **LAAO occlusion** | **OAT** | **p** | **SMD** | **LAAO occlusion** | **OAT** | **p** | **SMD** |
| n | 89 | 114 |  |  | 96.3 | 106.3 |  |  |
| Gender = Male (%) | 68 (76.4) | 75 (65.8) | 0.136 | 0.236 | 72.5 (75.2) | 71.3 (67.0) | 0.484 | 0.182 |
| Age (median [IQR]) | 74 [66, 80] | 76 [71, 80] | 0.096 | 0.293 | 74 [59, 79] | 73 [67, 79] | 0.569 | 0.221 |
| Age = >= 75 yrs (%) | 41 (46.1) | 64 (56.1) | 0.199 | 0.203 | 51.2 (53.1) | 47.2 (44.4) | 0.538 | 0.176 |
| Dialytic age= >= 3 yrs (%) | 34 (38.2) | 66 (57.9) | 0.008 | 0.402 | 43.9 (45.6) | 59.3 (55.7) | 0.506 | 0.204 |
| Hypertension = yes (%) | 79 (88.8) | 95 (83.3) | 0.371 | 0.157 | 79.1 (82.1) | 89.6 (84.3) | 0.817 | 0.057 |
| Diabetes Mellitus = yes (%) | 31 (34.8) | 36 (31.6) | 0.735 | 0.069 | 45.4 (47.1) | 45.3 (42.6) | 0.769 | 0.092 |
| Dyslipidemia = yes (%) | 49 (55.1) | 45 (39.5) | 0.039 | 0.316 | 50.8 (52.8) | 55.2 (52.0) | 0.957 | 0.016 |
| Peripheral artery disease = yes (%) | 49 (55.1) | 83 (72.8) | 0.013 | 0.376 | 73.2 (76.0) | 78.1 (73.5) | 0.779 | 0.058 |
| Ischemic cardiac disease = yes (%) | 42 (47.2) | 56 (49.1) | 0.895 | 0.039 | 43.2 (44.8) | 42.7 (40.2) | 0.753 | 0.094 |
| Heart failure = yes (%) | 30 (33.7) | 49 (43.0) | 0.23 | 0.192 | 47.8 (49.6) | 35.5 (33.4) | 0.238 | 0.334 |
| Ischemic stroke = yes (%) | 9 (10.1) | 12 (10.5) | 1 | 0.014 | 10.0 (10.4) | 8.8 (8.3) | 0.682 | 0.072 |
| Chronic pulmonary disease = yes (%) | 18 (20.2) | 21 (18.4) | 0.885 | 0.046 | 32.2 (33.4) | 21.4 (20.2) | 0.359 | 0.303 |
| Thromboembolic pulmonary disease = yes (%) | 2 (2.2) | 1 (0.9) | 0.829 | 0.111 | 1.3 (1.3) | 0.6 (0.5) | 0.455 | 0.082 |
| Bleeding = yes (%) | 56 (62.9) | 15 (13.2) | <0.001 | 1.194 | 32.2 (33.4) | 38.4 (36.1) | 0.838 | 0.056 |
| Type of AF (%) |  |  | <0.001 | 0.75 |  |  | 0.499 | 0.256 |
| Paroxysmal AF | 34 (38.2) | 12 (10.5) |  |  | 17.2 (17.9) | 9.8 (9.3) |  |  |
| Persistent AF | 14 (15.7) | 42 (36.8) |  |  | 24.1 (25.0) | 31.0 (29.1) |  |  |
| Permanent AF | 41 (46.1) | 60 (52.6) |  |  | 55.0 (57.1) | 65.5 (61.6) |  |  |
| Left ventricular hypertrophy = yes (%) | 43 (48.3) | 67 (58.8) | 0.18 | 0.211 | 59.5 (61.7) | 64.8 (61.0) | 0.953 | 0.015 |
| Left ventricular ejection fraction <50% = yes (%) | 17 (19.1) | 28 (24.6) | 0.448 | 0.132 | 29.3 (30.4) | 19.6 (18.4) | 0.385 | 0.282 |
| CHA2DS2VASc Score (median [IQR]) | 4 [3, 5] | 4 [4, 5] | 0.001 | 0.511 | 4 [3, 5] | 4 [3, 5] | 0.774 | 0.008 |
| HASBLED Score (median [IQR]) | 4 [4, 5] | 4 [3, 5] | 0.007 | 0.358 | 4 [3, 4] | 4 [3, 5] | 0.222 | 0.35 |
| Antiplatelet = yes (%) | 57 (64.0) | 32 (28.1) | <0.001 | 0.774 | 31.5 (32.7) | 39.1 (36.7) | 0.742 | 0.086 |
| Heparin = yes (%) | 29 (32.6) | 33 (28.9) | 0.686 | 0.079 | 43.3 (44.9) | 24.6 (23.1) | 0.094 | 0.474 |

**Supplementary Table 2: LAA occlusion versus No Therapy cohort**

|  | **Unweighted cohorts** | | | | **Weighted cohorts** | | | |
| --- | --- | --- | --- | --- | --- | --- | --- | --- |
|  | **LAA occlusion** | **No Therapy** | **p** | **SMD** | **LAA occlusion** | **No Therapy** | **p** | **SMD** |
| n | 89 | 148 |  |  | 76.7 | 154.5 |  |  |
| Gender = Male (%) | 68 (76.4) | 83 (56.1) | 0.003 | 0.44 | 49.7 (64.8) | 100.1 (64.8) | 0.996 | 0.001 |
| Age (median [IQR]) | 74 [66, 80] | 76 [69, 82] | 0.025 | 0.298 | 74 [67, 79] | 75 [62, 81] | 0.822 | 0.007 |
| Age = >= 75 yrs (%) | 41 (46.1) | 85 (57.4) | 0.118 | 0.229 | 34.2 (44.6) | 71.3 (46.2) | 0.864 | 0.031 |
| Dialytic age= >= 3 yrs (%) | 34 (38.2) | 66 (57.9) | 0.008 | 0.402 | 45.7 (59.6) | 93.3 (60.4) | 0.934 | 0.016 |
| Hypertension = yes (%) | 79 (88.8) | 131 (88.5) | 1 | 0.008 | 61.3 (79.9) | 137.4 (88.9) | 0.19 | 0.25 |
| Diabetes Mellitus = yes (%) | 31 (34.8) | 50 (33.8) | 0.981 | 0.022 | 24.5 (32.0) | 48.2 (31.2) | 0.919 | 0.017 |
| Dyslipidemia = yes (%) | 49 (55.1) | 41 (27.7) | <0.001 | 0.578 | 29.6 (38.6) | 52.4 (33.9) | 0.581 | 0.097 |
| Peripheral artery disease = yes (%) | 49 (55.1) | 101 (68.2) | 0.057 | 0.274 | 44.1 (57.5) | 86.4 (55.9) | 0.865 | 0.032 |
| Ischemic cardiac disease = yes (%) | 42 (47.2) | 75 (50.7) | 0.7 | 0.07 | 28.3 (36.8) | 70.8 (45.8) | 0.304 | 0.183 |
| Heart failure = yes (%) | 30 (33.7) | 54 (36.5) | 0.77 | 0.058 | 24.2 (31.5) | 50.2 (32.5) | 0.903 | 0.021 |
| Ischemic stroke = yes (%) | 9 (10.1) | 9 (6.1) | 0.378 | 0.148 | 12.3 (16.1) | 9.4 (6.1) | 0.066 | 0.322 |
| Chronic pulmonary disease = yes (%) | 18 (20.2) | 30 (20.3) | 1 | 0.001 | 13.8 (18.0) | 27.5 (17.8) | 0.976 | 0.005 |
| Thromboembolic pulmonary disease = yes (%) | 2 (2.2) | 5 (3.4) | 0.919 | 0.068 | 5.2 (6.8) | 3.4 (2.2) | 0.188 | 0.224 |
| Bleeding = yes (%) | 56 (62.9) | 36 (24.3) | <0.001 | 0.845 | 37.7 (49.2) | 68.0 (44.0) | 0.587 | 0.104 |
| Type of AF (%) |  |  | <0.001 | 0.779 |  |  | 0.416 | 0.251 |
| Paroxysmal AF | 34 (38.2) | 43 (29.1) |  |  | 28.7 (37.4) | 43.6 (28.2) |  |  |
| Persistent AF | 14 (15.7) | 72 (48.6) |  |  | 22.2 (28.9) | 61.8 (40.0) |  |  |
| Permanent AF | 41 (46.1) | 33 (22.3) |  |  | 25.8 (33.7) | 49.1 (31.8) |  |  |
| Left ventricular hypertrophy = yes (%) | 43 (48.3) | 82 (55.4) | 0.355 | 0.142 | 42.8 (55.8) | 87.2 (56.4) | 0.939 | 0.014 |
| Left ventricular ejection fraction<50% = yes (%) | 17 (19.1) | 38 (25.7) | 0.316 | 0.158 | 13.7 (17.8) | 31.3 (20.3) | 0.718 | 0.062 |
| CHA2DS2VASc Score (median [IQR]) | 4 [3,5] | 5 [3,6] | 0.001 | 0.454 | 4 [3,5] | 4 [3,5] | 0.894 | 0.008 |
| HASBLED Score (median [IQR]) | 4 [4,5] | 4 [4.5] | 0.225 | 0.156 | 4 [3,5] | 4 [3,5] | 0.946 | 0.024 |
| Antiplatelet = yes (%) | 57 (64.0) | 104 (70.3) | 0.395 | 0.133 | 45.7 (59.6) | 93.3 (60.4) | 0.934 | 0.016 |
| Heparin = yes (%) | 29 (32.6) | 30 (20.3) | 0.049 | 0.282 | 25.0 (32.6) | 35.2 (22.8) | 0.232 | 0.221 |

**Supplementary Table 3: Weighted Cox model on hemorrhagic events, overall mortality and cardiovascular events at two years of follow-up.** Hemorrhagic events are evaluated in the first three months and in the following 21 months from procedure (p-value for treatment and time interaction were 0.330 for OAT vs LAA occlusion and 0.048 for No Therapy vs LAA occlusion, respectively)

| **OAT vs LAA occlusion** | **HR** | **95%CI** | **p value** |
| --- | --- | --- | --- |
| Hemorrhagic events (1-3 months) | 2.03 | 0.37-11.07 | 0.414 |
| Hemorrhagic events (4-24 months) | 6.24 | 1.36-28.62 | 0.018 |
| Overall mortality | 3.76 | 1.79-7.92 | <0.001 |
| Cardiovascular events | 3.79 | 1.61-8.95 | 0.002 |

| **No-Therapy vs LAA occlusion** | **HR** | **95%CI** | **p value** |
| --- | --- | --- | --- |
| Hemorrhagic events (1-3 months) | 0.56 | 0.16-1.94 | 0.359 |
| Hemorrhagic events (4-24 months) | 4.29 | 0.88-20.99 | 0.072 |
| Overall mortality | 2.80 | 1.44-5.42 | 0.002 |
| Cardiovascular events | 3.50 | 1.80-6.75 | <0.001 |

LAA: Left Atrial Appendage; OAT: Oral Anticoagulant Therapy

**Supplementary Table 4: Cox model on hemorrhagic events, overall mortality and cardiovascular events at two years of follow-up excluding peritoneal patients (n=90). Hemorrhagic events are evaluated in the first three months and in the following 21 months from procedure.**

|  | **Univariate analysis** | | | **Multivariate analysis*** | | |
| --- | --- | --- | --- | --- | --- | --- |
| **OAT vs LAA occlusion** | **HR** | **95%CI** | **p-value** | **HR** | **95%CI** | **p-value** |
| Hemorrhagic events (1-3 months) | 0.59 | 0.18-1.94 | 0.386 | 1.60 | 0.42-6.09 | 0.493 |
| Hemorrhagic events (4-24 months) | 3.29 | 0.73-14.71 | 0.120 | 6.24 | 1.27-30.60 | <0.001 |
|  |  | |  | *Time-interaction p-value:* | | 0.199 |
| Overall mortality | 2.14 | 1.16-3.95 | 0.015 | 2.71 | 1.28-5.73 | 0.009 |
| Cardiovascular events | 2.70 | 1.58-4.64 | <0.001 | 4.96 | 2.45-10.06 | <0.001 |

|  | **Univariate analysis** | | | **Multivariate analysis*** | | |
| --- | --- | --- | --- | --- | --- | --- |
| **No-Therapy vs LAA occlusion** | **HR** | **95%CI** | **p-value** | **HR** | **95%CI** | **p-value** |
| Hemorrhagic events (1-3 months) | 0.54 | 0.17-1.68 | 0.288 | 0.57 | 0.18-1.81 | 0.337 |
| Hemorrhagic events (4-24 months) | 3.55 | 0.81-15.50 | 0.093 | 4.75 | 1.05-21.42 | 0.042 |
|  |  | |  | *Time-interaction p-value:* | | 0.029 |
| Overall mortality | 2.31 | 1.27-4.19 | 0.006 | 3.01 | 1.56-5.81 | 0.001 |
| Cardiovascular events | 2.41 | 1.41-4.10 | 0.001 | 3.03 | 1.74-5.28 | <0.001 |

LAA: Left Atrial Appendage; OAT: Oral Anticoagulant Therapy. * adjusted for gender, age, dialytic age, CHA2DS2VASc,

HASBLED, type of atrial fibrillation, dyslipidemia, peripheral artery disease, previous bleeding, antiplatelet,

**Supplementary Table 5: Post-procedural therapies prescribed to patients undergoing left atrial appendage occlusion**

|  | **N** | **%** |
| --- | --- | --- |
| Aspirin + Clopidogrel | 57 | 62.0 |
| Aspirin | 10 | 10.9 |
| Clopidogrel | 9 | 9.8 |
| Heparin | 3 | 3.3 |
| Aspirin + Heparin | 3 | 3.3 |
| Ticlopidine | 2 | 2.2 |
| Clopidogrel + Heparin | 2 | 2.2 |
| Aspirin + Vitamin K Antagonist | 1 | 1.1 |
| Aspirin + Ticlopidine | 1 | 1.1 |
| Clopidogrel + Vitamin K Antagonist | 1 | 1.1 |
| Aspirin + Clopidogrel + Heparin | 1 | 1.1 |
| No therapy | 2 | 2.2 |
